# Understanding Serial Mediators of Problematic Pornography Use in Pakistani Men and Women

**DOI:** 10.3390/ijerph192114336

**Published:** 2022-11-02

**Authors:** Khifza Bibi, Ambreen Fatima, Rizwana Amin, David L. Rowland

**Affiliations:** 1 Department of Professional Psychology, Bahria University, Islamabad 44000, Pakistan; 2 Department of Psychology, Valparaiso University, Valparaiso, IN 46383, USA

**Keywords:** pornography use, addiction, coping, inhibitory control, predisposing factors, mediating factors, craving, developing nations

## Abstract

In the current digital environment, satisfying sexual needs via Internet pornography use has the potential to develop into a problem that affects one’s psychological health and daily functioning. The aim of this study was to examine potential cognitive and affective factors that could help explain the maintenance and exacerbation of self-defined problematic internet pornography use. Methods: 280 Pakistani men and women (mean age = 25.40; SD = 5.271, range 18–50) who were current pornography users were recruited through social networking sites (Facebook, Instagram, LinkedIn, WhatsApp groups) to participate in an online study about pathways to problematic pornography use (PPU). Structural equation modeling was used to estimate path analysis coefficients extending from predisposing variables (depression, anxiety, self-esteem, and loneliness) to PPU via the mediating variables of craving, dysfunctional sexual coping, and stimulus-specific inhibitory control. Results: Craving mediated the relationship between three predisposing variables (depression, anxiety, and self-esteem) and PPU, though not the fourth, namely loneliness. Indirect effects of depression, anxiety, and self-esteem were significantly linked to PPU through two serial mediation pathways: (a) craving and stimulus-specific inhibitory control, and (b) craving and dysfunctional sexual coping. Conclusion: These findings demonstrate that craving, stimulus-specific inhibitory control, and dysfunctional coping serve as important mediators in maintaining and exacerbating the cycle between negative predisposing variables and PPU. These results are interpreted within the general framework of therapeutic interventions that can help develop positive coping skills in individuals seeking to alter self-perceived bothersome or unwanted habits related to pornography use.

## 1. Introduction

The Internet has enabled greater accessibility to information and content to worldwide populations than has ever been known before—an effect that offers great advantages in some areas, yet significant problems in others, e.g., trafficking, scams, cyberbullying, and so on [1]. Pornographic content is one area that, although once highly controlled through socio-cultural restrictions and laws [2], has now become increasingly accessible through the Internet, even in world regions that censure/block such sites (e.g., [3,4]). Indeed, in some parts of the world, pornography use has become commonplace; for example, a probability sample from the USA indicates that 94% of males and 87% of females have used pornography at some point in their lifetime [5]. In other parts of the world, pornography use is reaching all-time highs [4].

The effect of pornography on the individual and society is the subject of controversy, partly because the issue is often imbued with strong ideological stances, but also because well-designed multivariate studies are often lacking. Pornography use purportedly has a number of positive outcomes, such as sex educational benefits for the uninformed; as a way to enhance sexual pleasure for couples; and as a means for overcoming pathophysiological or psychological obstacles to sexual arousal [6,7]. However, negative effects have also been reported, including the potential for sexual aggression and increased risky sexual behavior through emulation of pornographic characters and activities, possible victimization of (especially women) actors, and becoming overly dependent on pornographic content to achieve sexual arousal during masturbation (but see [8]).

### 1.1. Factors Relevant to Pornography Use

The conditions that enable frequent and/or excessive pornography use are now more prevalent than ever and, as a result, some of the consequences of pornography use—positive and negative—are becoming ever more apparent. Specifically, the removal of traditional barriers to pornography use has increased availability, accessibility, and anonymity [9], increasing both its probability and frequency, and concomitantly increasing vulnerability for habitual use. Furthermore, given the sexually reinforcing experiences often associated with pornography use, the motivation to consume pornography may increase over time, even reaching a point of excess. Very frequent or excessive pornography use may not only become necessary for sexual arousal [10] but may even encroach upon normal daily functioning and psychological health [11,12,13], representing conditions characteristic of a behavioral addiction. (Some sources distinguish between chemical addictions and behavioral addictions, preferring the terminology compulsive behavior rather than addiction. Beyond substance addiction, DSM-V currently considers gaming as the only psychological/behaviorally based addiction. [14]).

However, increases in pornography use and frequency are not merely the consequence of increased availability and anonymity. Given the wide individual variation in pornography use, numerous studies have attempted to understand why some individuals seem more vulnerable to developing problematic pornography use (PPU) than others [15,16,17,18]. Several models have attempted to account for this variation—and in alignment with more general models of addictive/compulsive behavior—they typically posit the existence of *predisposing factor*s that increase an individual’s vulnerability to addiction in general, in conjunction with *immediate* and *proximal situational* stimuli that account for the specific occurrences of the addictive behavior [19,20,21,22].

#### 1.1.1. Predisposing Factors

Brand et al. [22] proposed a two-stage model of addiction known as I-PACE (Interaction of Person, Affect, Cognition, Execution), with the early stage explained by pre-dispositional factors (i.e., “Person” in the model). These include genetic tendencies, early childhood experiences, psychopathology, and temperamental features and behaviors (self-esteem, loneliness) that predispose the individual not only for general addictive potential but also for motives toward specific types of addictions (Internet gaming, gambling, shopping, social media communication, pornography use, etc.).

Indeed, various studies have supported a link between predisposing psychopathological factors (e.g., depression or anxiety) and PPU [23,24,25,26,27]. Various personality characteristics have also been associated with PPU, including inadequate social skills (e.g., lack of assertive skills), low self-esteem, lack of self-efficacy [28,29,30], and loneliness [31,32]. Most research designs to date, however, have been unable to specify the order of relationships for these variable links.

#### 1.1.2. Immediate and Proximal Factors

As noted above, predisposing (Person) characteristics presumably set the stage for the later phase of the development of addictive behavior (as suggested by the I-PACE model) by interacting with ongoing situational variables that trigger Affective and Cognitive responses (e.g., cue reactivity, craving, urge for mood regulation, attentional bias, etc.) that culminate in the decision to Execute specific behaviors (e.g., gaming, pornography use). These specific behaviors result in gratification and/or suppression of negative affect, providing reinforcing effects which, through operant conditioning, lead to the development of reactivity to and craving for the associated triggering cues. As a consequence, the likelihood of executing the specific behavior in the future increases. Included in the I-PACE model are various moderating and mediating factors. For example, general inhibitory control and/or executive functioning may act as moderators in the early stages of addiction development whereas dysfunctional coping style and (lack of) stimulus-specific inhibitory control may mediate the relationship between affective/cognitive responses and the addictive behavior in the later stages. Such processes have been demonstrated in studies delineating, for example, the important role of sexual arousal and craving responses specific to pornography addiction [17,18,26,33,34].

### 1.2. I-PACE Model and Pornography Use

Although pornography use and sexual engagement may in some instances represent a beneficial strategy for alleviating anxiety [35], individuals who come to rely on pornography use to regulate their negative emotional states are typically manifesting a dysfunctional coping strategy [18,21]. Furthermore, I-PACE suggests that individuals who use pornography with high frequency with the cognitive expectancy of both gaining pleasure and suppressing their everyday worries and responsibilities (i.e., especially to the point of disrupting daily functioning) are at higher risk for PPU and addiction [11,21,36]. Accordingly, people with PPU often report a lack of inhibitory control, consistent with the idea that the loss of control of executive functioning may be involved in the early stages of addiction, that is, at a time when the conditioned response to pornography stimulation is first developing [37]. For these individuals, it becomes increasingly difficult to control their addictive tendency, despite the concomitant negative repercussions of their behavior (e.g., disrupting daily functioning). The model further suggests that as the problematic behaviors become increasingly entrenched, *stimulus-specific inhibitory control* in response to explicit (i.e., pornographic material) or implicit (pornographic ideation) cues is greatly reduced. Thus, increasingly frequent pornography use can involve elements of both operant and classical conditioning, not only with respect to overt behavioral responses, but also regarding covert emotional responding [13,38].

## 2. Context, Rationale, and Aims

Within Pakistani and many other Eastern cultures, it is generally considered taboo to express ideas or self-disclosures about sexuality [39], except within heterosexual marital boundaries [40]. In 2019, Pakistan Telecommunication Authority banned more than 800,000 pornography websites on the basis of legal repercussions and presumed negative psychological and emotional consequences [41], thus ranking Pakistan as the top country to ban such websites. Despite these cultural and legal restrictions, the number of Pakistani engaging in cyber sexual activities is still high. For example, 67.5% of 16–24-year-olds from urban areas of Pakistan reported regularly viewing pornographic content [39]. This rate compares with another recent Asian subcontinent study from India in which 54% of respondents reported Internet pornography use, 11% of whom were problematic users [42], and also with a recent Malaysian study on college students that found that over 74%—mostly males—reported regular exposure to pornography [43].

The current study was aimed at testing and expanding upon the I-PACE model within the context of a non-Western sample where open discussion of issues surrounding sexuality is generally taboo or prohibited. Specifically, we investigated ongoing cognitive and affective factors involved in maintaining and/or intensifying regular/frequent (often problematic) pornography use in individuals who already had at least some experience with pornographic content, as such individuals have presumably experienced gratifying outcomes related not only to sexual arousal/reward but also to attenuation of negative emotional states. Such pornography use would typically be deemed “problematic” not only when an individual perceives a lack of self-regulation of their behavior, but also—in highly restrictive religious cultures—when the individual experiences discomfort/anxiety due to the moral incongruence between their actual vs. expected behaviors [44].

**Figure 1:**
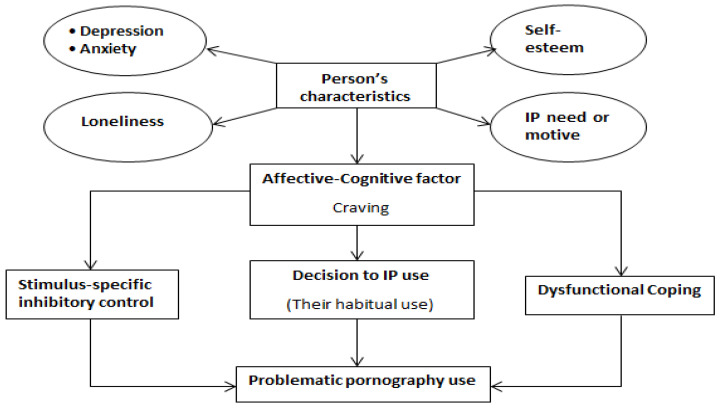
Integrated conceptual model of the study.

To better understand the interrelationships between the above cognitive-affective associations and specific behavioral executions, our methodology utilized a broad multivariate SEM approach that not only controlled for collinearity of variables, but also enabled sequencing of variables so as to strengthen the ordering of relationships within the model. Such “mediation” approaches enable the researcher to determine how much of the association between X and Y is the result of an intervening (mediating) variable, M. Although prior research has explored mediating and moderating variables for a variety of addictive behaviors, the current study attempted to elaborate the pathway from predisposing variables (e.g., psychopathological, personality, and social cognitive) to PPU through serial mediation analysis that included critical explanatory variables such as pornography craving, stimulus-specific inhibition, and dysfunctional coping (see Figure 1). Although such variables have previously been implicated (though not thoroughly tested) in the development of PPU [19,37], instead of including *general* inhibitory control and coping, we assessed variables more specific to sexual addiction using an instrument designed to assess both (a) the lack of stimulus-specific inhibitory control related to pornographic content and (b) a dysfunctional coping strategy specifically related to sexual arousal (i.e., the tendency to use sex in response to elevated negative mood states or life stressors) [38].

To this end, we hypothesized that (see Figure 1): (H1) predisposing variables (psychopathology; depression and anxiety, self-esteem and loneliness) have significant associations with PPU, with pornography craving serving as a mediator; (H2) predisposing variables are significantly associated with PPU, with pornography craving and *dysfunctional coping* as *serial* mediating variables; and (H3) predisposing variables are significantly associated with PPU, with pornography craving and *stimulus-specific inhibitory control* as *serial* mediating variables.

## 3. Materials and Methods

### 3.1. Participants

This cross-sectional web-based study recruited participants through social media (see Procedure section) who were English-language competent (English is one of two official languages in Pakistan). Initially, 297 individuals responded over a 4 months period; of these 94% met the inclusion criteria; 6% (n = 17) were excluded because of incomplete information, because they did not use pornographic materials, or because they were not Pakistani nationals. The final sample consisted of 280 Pakistani participants, 123 women and 157 men, (mean age = 25.40; SD = 5.271, range 18–50).

### 3.2. Questionnaires and Survey Instruments

Assessment forms and instruments are presented below, with sample items of each provided in Appendix A. Each instrument is identified as assessing a predisposing, mediating, or outcome variable.

#### 3.2.1. Demographic Form (Screening Form)

Participants provided their age, self-identified gender, and primary sexual orientation (bisexual, homosexual/gay/lesbian, or heterosexual). Three brief questions also inquired about their pornography use, including the duration and frequency, as well as perceived gratification from use. These items were ordinally scaled and therefore could not be included in the SEM analysis, but along with other variables of interest are explored in a separate paper.

#### 3.2.2. Depression Anxiety and Stress Scale-21 (DASS-21) (Predisposing Variables)

The DASS-21 includes three subscales: depression, anxiety, and stress [45]. Each subscale represents the sum of its 7 individual items, with responses ranging from never (0) to almost always (3). For the current study, depression and anxiety subscales were used to assess psychopathological symptomology. Recommended cut-off scores for conventional severity labels for the depression and anxiety scales were: normal (0–9, depression) (0–7, anxiety) to extremely severe (28+, depression) (20+, anxiety). DASS-21 has strong overall reliability (α = 0.91), and coefficients for the depression and anxiety subscales were 0.81 and 0.89, respectively.

#### 3.2.3. Rosenberg Self-Esteem Scale (Predisposing Variable)

Rosenberg Self-Esteem Scale [46] consists of 10 items with responses ranging from strongly agree (1) to strongly disagree (4). Lower self-esteem is indicated by responses of “disagree” or “strongly disagree” on items 1, 3, 4, 7, and 10, and “strongly agree” or “agree” on items 2, 5, 6, 8, and 9. The scale is scored by totaling the individual 4-point items after reverse-scoring items 2,5,6,8, and 9. Higher scores represent higher self-esteem. The scale has strong internal reliability, with α = 0.92.

#### 3.2.4. The 6-Item De Jong Gierveld Loneliness Scale (Predisposing Variable)

The 6-Item De Jong Gierveld Loneliness Scale [47] is an abbreviated version of the 11-item scale of the same name. This scale has two subscales, emotional loneliness and social loneliness, each consisting of 3 items with response options of “Yes,” or “Yes, more or less,” both of which were assigned a value of 1, and “No,” assigned a value of 0. A total score is computed by adding the emotional and social loneliness scores. The total score is valid only when missing or unanswered questions do not exceed 1, that is, if the sum of the missing emotional loneliness score and the missing social loneliness score equals zero or one. Cronbach’s α is 0.76 for emotional loneliness and 0.86 for social loneliness. Summed scores range from 0 to 6, with 0 indicating the least loneliness.

#### 3.2.5. Pornography Craving Questionnaire (PCQ) (Mediating Variable)

To measure pornography craving, PCQ was used. PCQ is a 12-item measure [48] with 7 response options ranging from 1 (disagree completely) to 7 (agree completely). Item 1 measures control over pornography use, Items 9 and 12 measure current desire to use pornography, Items 2 and 3 measure psychophysiological reaction to pornography use, Items 6,7, and 10 measure intention to watch pornography, and Items 4,5,8, and 11 measure mood change after watching pornography. A mean score can be calculated from the 12 items, with a possible range of 1 to 7. A score of 5.0 or greater is considered “positive” for craving. The scale has good overall internal reliability, α = 0.89.

#### 3.2.6. Hypersexual Behavior Inventory-19 (HBI-19) (Mediating Variables)

Hypersexual Behavior Inventory-19 [38] was used in the present study to identify dysfunctional coping related to sexual urges and stimulus-specific inhibitory control. HBI-19 has three domains: coping, consequences, and control, scaled 1 to 5. Only the Coping (7 items) and Control (8-items) subscales were considered relevant for this study, with domain-specific scores obtained by summing the respective items. The scale has good validity and overall internal reliability (α = 0.96), as do the subscales (Control, α = 0.95; Coping, α = 0.91).

#### 3.2.7. Brief Pornography Screening (BPS) (Outcome Variable)

The Brief Pornography Screening scale was used to identify problematic pornography use (PPU) [49]. BPS is a 5-item measure with three response options ranging from 0 (never) to 2 (very often), with a score of 6 or more reflecting the possible need for clinical help or assistance. As the score reflects a self-perceived “problem” with pornography use, a high score may be influenced by part by dissatisfaction with oneself based on perceived deviation from cultural norms. The scale has a high reliability (α = 0.89).

### 3.3. Procedure

Ethical approval was obtained from the Institutional Review Board (IRB) of the first author’s institution. A brief pilot study determined that likely participants could understand English versions of the assessment forms, as nearly all showed strong comprehension due to the simple lexicon of the instruments.

The study was promoted by creating a video on Facebook to encourage people to express their opinions about issues related to pornography. It was posted on other free social networking sites, including Facebook, WhatsApp, Instagram, and LinkedIn groups, with each providing a link to the online (web-based) survey (a recruitment script is provided in Appendix B). When potential respondents followed the link, they were informed about the purpose of the study and assured anonymity, as no individual identifying information was collected.

Candidates were then screened with the question: “Do you watch adult-themed/pornographic content,” and those responding “yes” were able to proceed to the next step, in which they gave anonymous consent by responding affirmatively to the statement “I hereby agree to take part in the study as a participant.” They were then invited to complete all the assessment forms, and upon submission, they were warmly thanked for their time and information.

Best practices were followed in the design and execution of the survey [50]. For example, anonymity was guaranteed, no rewards or prizes were offered, internally consistent response patterns were verified by generating Cronbach alpha values, and using Google survey settings, only one-time submission was permitted. In addition, completion of the questionnaires required about 20 min, and in order to proceed from one question to the next, each question required a response. As such, all 280 participants included in the final sample generated a full complement of data.

### 3.4. Statistical Analysis

Descriptive analysis and alpha coefficients were calculated using SPSS (IBM Corp. Released 2017. IBM SPSS Statistics for Windows, Version 25.0. Armonk, NY, USA: IBM Corp.), and structural equation modeling (SEM) used AMOS (version 23). Before testing the SEM, the fits for the measurement model and confirmatory factor analysis (CFA) were also tested in AMOS. For both the CFA and SEM, maximum likelihood parameter estimation was applied. For the evaluation of standard criteria of good fit, indices for both CFA and SEM were applied [51]. Specifically, a non-significant chi square (χ^2^) indicated that the model fit the data well, along with the following: comparative fit indices (CFI)/Tucker–Lewis Index (TLI) > 0.90 (acceptable fit); and CFI/TLI > 0.95 (good fit); and Root Mean Square Error of Approximation (RMSEA) < 0.08 (good fit), and RMSEA = 0.08–0.10 (acceptable fit).

## 4. Results

Table 1 provides a description of the sample. Descriptive statistics and alpha coefficients for the study variables appear in Table 2. Table 3 provides Pearson correlation coefficients among variables which, although ranging from weak to strong, met the SEM prerequisites of significance. Correlation values exceeding 0.50 (e.g., 0.50–0.65) indicated that while several measures were likely assessing related constructs, they were not redundant.

### 4.1. Preliminary Exploration of Sex and Orientation Differences

Preliminary analysis indicated no sex/gender differences on the major study variables in the I-PACE model (*p* ≥ 0.435) (Table 4). In addition, chi-square indicated that the distribution of gay/lesbian and bisexual participants was distributed fairly evenly across the two sexes (χ^2^ = 0.395, *p* = 0.821), 9.6% men, 10.5% women for gay/lesbian; and 26.0% men, 29.3% women for bisexual. Consequently, participants of both sexes were included in each of the two models described below.

### 4.2. Model Development for Path Analysis

As iterated in the study aims, we developed two statistical models based on I-PACE [22], the first with stimulus-specific inhibitory control as a mediating variable, and the second with dysfunctional sexual coping as a mediating variable (refer back to Figure 1).

**Figure 2:**
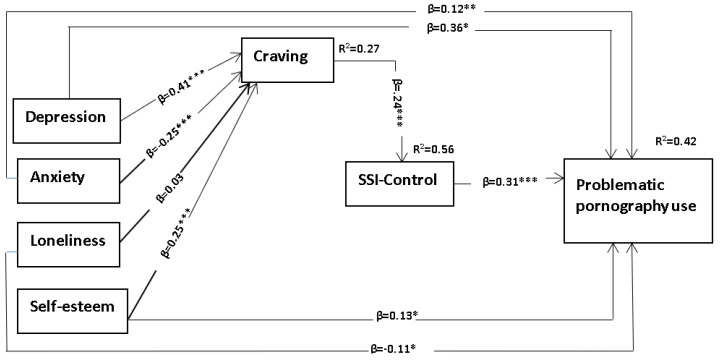
Results of the structural equation model of Model 1 including β-weights, p-values, and residuals. Note: SSI-Control = stimulus-specific inhibitory control, β = standardized regression coefficients, R^2^ = Squared multiple correlation, level of significance; * *p* < 0.050; ** *p* < 0.010; *** *p* < 0.001.

Model 1 (Figure 2) tests the idea that “craving”—established through initial pornography use and presumed subsequent gratification—in the presence of specific ongoing “Person” (predisposing) variables (such as low self-esteem, loneliness, depression, anxiety) increases PPU through the serial mediating variable of (lack of) stimulus-specific inhibitory control (e.g., pornographic ruminations or actual pornographic content).

**Figure 3:**
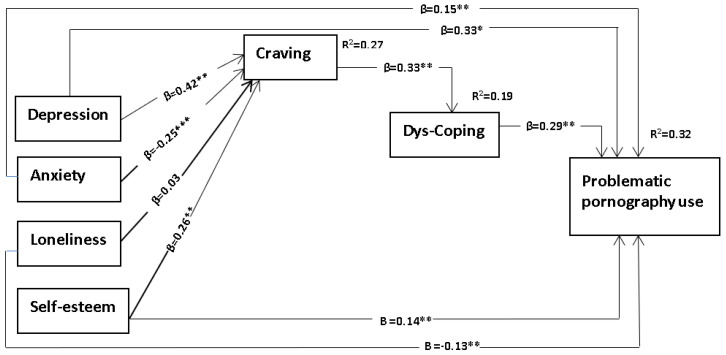
Results of the structural equation model of Model 2 including β-weights, p-values, and residuals. Note: Dys-coping = dysfunctional coping, β = standardized regression coefficients, R2 = Squared multiple correlation, level of significance; * *p* < 0.050; ** *p* < 0.010; *** *p* < 0.001.

Model 2 (Figure 3), using the same basic premises as Model 1, tests the idea that “craving,” in the presence of specific ongoing “Person” (predisposing) variables (such as low self-esteem, loneliness, depression, anxiety) increases PPU through the serial mediating variable of dysfunctional sexual coping strategies.

Thus, two models were estimated, one with stimulus-specific inhibitory control and the other with dysfunctional coping as a mediating variable, using 2000 bootstrap samplings from the given dataset for generating indirect effects. The 95% bias-corrected confidence intervals (CIs 95%) and user-defined estimands were used to estimate the significance of defined paths as shown in Table 5.

Prior to calculating serial mediation analysis, simple mediation with “craving” as the only mediator was estimated. Three of the four pathways from predisposing factors (depression, anxiety, and self-esteem) to craving were significant (*p* ≤ 0.002) (Table 5). The overall effect of loneliness on problematic pornography use was significant (β = 0.13; *p* < 0.05). However, the loneliness-craving-pornography pathway was not significant (β = 0.01; *p* = 0.59) and therefore loneliness was removed from further serial analysis. Then, the two proposed models (mentioned above), with the added serial mediation factors (stimulus-specific inhibitory control, and dysfunctional coping) were estimated so as to determine their fit with the data. Results indicated that both models met the aforementioned model fit criteria well.

Specifically, Model 1 with stimulus-specific inhibitory control indicated a good fit with the data ꭓ^2^ = 6.24 (df = 3), *p* = 0.10; CFI = 0.99, TLI = 0.95; RMSEA = 0.062; and SRMR = 0.023. Path analysis confirmed the hypothesized relationships that the indirect association of depression, anxiety, and self-esteem on PPU significantly flowed through serial mediation of craving and stimulus-specific inhibitory control (Table 5, Figure 2).

Model 2, with dysfunctional coping, also indicated a good fit with the data, ꭓ^2^ = 6.34 (df = 3), *p* = 0.096, CFI = 0.99, TLI, 0.94, RMSEA 0.063 and SRMR 0.022. Specifically, the indirect association of depression, anxiety, and self-esteem on PPU flowed through craving and dysfunctional coping (Table 5, Figure 3).

## 5. Discussion

This study identified factors that mediate Person (predisposing) characteristics (psychopathological, loneliness and low self-esteem) on PPU in men and women pornography users in Pakistan. Our aim was to specify the mediating pathways of the general Internet addiction model (I-PACE: [37]) specifically with respect to understanding PPU within a non-Western culture. Pornography craving, dysfunctional coping, and stimulus-specific inhibitory control were examined either as simple mediating variables or as serial mediating variables. Both pornography craving and self-identified PPU were fairly high in our sample, with over 80% indicating a “positive” craving as defined by the assessment scale, and about 70% indicating a level of PPU suggesting possible need for help or clinical assistance.

### 5.1. Explanation of Factors Mediating the Relationship between Predisposing Factors and PPU

Our first hypothesis—that “craving” mediates the relationship between predisposing variables and PPU—was supported for three of the four predisposing variables: depression, anxiety and self-esteem (see also, [52]). In other words, the relationship between these predisposing factors and PPU became significant only when “craving” was introduced as a mediating variable. Thus, these predisposing factors did not affect PPU directly, but rather did so only through “craving.” Such findings are not surprising, as craving is considered an important element for any type of addiction, and in our study, it correlated (r = 0.49) with self-defined PPU, suggesting that these two variables—though not redundant—were likely tapping into the same general construct. That is, the stronger the craving to view pornography, the more likely the participant viewed their pornography use as problematic—either because they felt it was out of control or because it was morally incongruent with religious/cultural expectations (or both). However, in contrast with prior research, loneliness (also a predisposing variable) showed a significant direct relationship with PPU, showing no significant link with craving (see also [32,53]). Consistent with this finding, previous studies elaborating upon the association between loneliness and pornography use have found that frequent users in Pakistan show low social engagement in general, presumably driven by their associated feelings of low self-esteem, shame, guilt, and frustration [54]. For these “lonely” users, craving did not act as a catalyst between loneliness and PPU—indeed, these lonely users may have used pornography as a substitute for their low level of social engagement.

Our second hypothesis tested the role of “dysfunctional sexual coping” as a mediating factor between craving and PPU. For the three relationships tested, dysfunctional coping served as a second mediator in the serial mediation from predisposing variables (depression, anxiety, and self-esteem) to the endpoint of self-defined PPU, with 32% of the variation in PPU explained by the constellation of predisposing and the two serial mediating factors (craving and dysfunctional coping). Specifically, the link between predisposing variables and PPU was sequentially mediated first through craving, and then through dysfunctional sexual coping (see also [52,55]).

Although not directly addressed in our study, the negative effects of craving on PPU could presumably be countered by a high level of functional coping [56], and taken together with our results, this information identifies a clear point and strategy for intervention for pornography users who view their habit—for whatever reason—as problematic. Specifically, even though these users may have developed sustained cravings for pornography, the development of functional coping tools to replace dysfunctional sexual coping strategies could help users develop a better sense of control over their problematic behavior. Because engagement in any unwanted behaviors (e.g., pornography use, overeating, drug use) is itself likely to exacerbate feelings of low self-esteem, anxiety, and depression [57], a greater sense of control over self-perceived “bad habits” is likely to raise the individual’s feeling of self-efficacy and, in doing so, help mitigate feelings of depression, anxiety, and low self-esteem, thereby helping to break the cycle between negative emotional states, craving, and PPU.

Our third hypothesis was also supported in that predisposing factors (depression, anxiety, and self-esteem) led to PPU via serial mediation of craving and (lack of) stimulus-specific inhibitory control. Specifically, 42% of the variation in PPU could be attributed to this constellation of predisposing variables and the two mediating effects. These findings reiterate the idea that compulsive pornography users often feel powerless to control their excessive usage, despite its negative repercussions [58]. The lack of control in our sample of pornography users fits well with the general notion that both craving *and* lack of control to specific cues are central and inevitable characteristics of behavioral [59] and substance addictions [60,61]. As with other behavioral addictions, PPU is strongly driven by associative learning processes, where pornographic stimuli and sexual gratification become firmly entrenched through classical conditioning and, as further elaborated by Brand [26], the perceived sexual gratification operantly reinforces the urge to re-visit of pornography sites. Users’ attempts to gain behavioral control may be further stymied by their own perceived lack of control over their “addiction,” part of a self-fulfilling mindset often a characteristic of addicts [20].

To reiterate, dysfunctional coping mediates the link between craving and PPU. Yet, at the same time, the lack of stimulus-specific inhibitory control also mediates this link. We note, however, that these are not unrelated processes. Not only were these constructs assessed via the same instrument (HBI-19), but our analysis showed that dysfunctional sexual coping correlated moderately with the lack of stimulus-specific inhibitory control. In this respect, these two constructs—dysfunctional sexual coping and stimulus specific control related to pornographic content—might be conceptualized as distinct components of the same construct.

### 5.2. Therapeutic Implications: Addressing PPU through Coping Strategies

Coping of any type typically involves active cognitive/behavioral attempts to either manage the stressful situation (problem-focused) or resolve/reduce the stress itself (emotion-focused), or both [62]. Problem-focused strategies identify factors responsible for the stressful situation and formulate ways to deal with them (including removing them). Thus, for men and women engaging in PPU, functional coping that involves management of the situation would include proactive attempts to remove or attenuate the effective triggering stimuli, or removing oneself from situations that mediate exposure to the stimuli. However, pornographic content is readily visualized and can persist in the absence of actual physical stimuli in the form of sexual imagination/fantasy. Nevertheless, although the associated cravings may be quite powerful, cognitive/behavioral therapeutic strategies can often alter such disruptive thought patterns by replacing them with positive ideations and actions, ones leading to alternate (more positive) outcomes while, at the same time, not necessarily excluding pathways to sexual gratification.

An important second strategy implicit in positive coping lies in the management of stress/emotions. As noted previously, people engage in compulsive/addictive behaviors not only because they lead to rewarding outcomes through positive feelings, but also because they can help suppress negative feelings such as anxiety, tension, low self-worth, and so on. Thus, emotion-focused coping represents a stress-management strategy with a focus on regulating the negative emotional reactions to a stressful situation, an approach that may be most practical when removing or attenuating the effective triggering stimuli is difficult or impossible. These adaptive strategies might include relaxation, mindfulness, pursuing social support and help, and cognitive reframing, strategies that have been linked to better self-esteem, reduced self-blame, greater emotional regulation, and lower depression [63]. Such intervention strategies represent a second therapeutic option for individuals feeling overwhelmed by powerful ideation associated with pornographic content and could well offer hope to individuals who experience significant distress due to any behaviors they view as detrimental (in the longer term) to psychological adjustment.

### 5.3. The Context of Non-Western Cultures

Although the above therapeutic strategies do not offer a panacea, the more tools individuals have at their disposal for addressing an unwanted behavior, the more support they have through social and psychological-health networks [64], and the more prepared they are for dealing with behavioral relapses, the greater the chance of making progress toward self-desired behavioral changes. Furthermore, such intervention strategies do not necessarily exclude the use of medications that might assist in the short-term regulation of negative emotional feelings and desires. At the same time, from the perspective of community psychological health in Pakistan and other developing geo-cultural regions, we recognize that there are other significant obstacles to rehabilitation, for example, the challenge of lowering barriers to seeking help not only by de-stigmatizing the use of mental health services, but also by increasing their affordability and availability to the general public, for example through university-based training clinics and online counseling services.

Finally, we note that, as expected [5,43], men and women pornography users in our sample did show differences in how long they have been using pornography, the typical duration of each pornography viewing, and the amount of sexual gratification they felt. For all three variables, men reported higher levels than women. These sex differences, although not included in the current analysis due to restrictions on including categorical or ordinal variables in SEM models, are the focus of subsequent analysis aimed at understanding independent predictors of pornography craving, coping, control, and problematic use. Despite these differences, however, the lack of sex differences on the constructs assessed in this analysis supports the general idea that the I-PACE model has broad application to the understanding of sustaining and perpetuating variables in the process of PPU, independent of the sex of the user and within widely differing cultures.

## 6. Study Strengths and Limitations

This study has demonstrated sequencing of variables leading to PPU and, in doing so, helps establish possible ordered links among variables. In fact, these pathways were able to account for 30–40% of the variation in PPU in a model that included both men and women. The fact that these findings were evident in a non-Western sample helps support the validity of I-PACE conceptualization to populations of varied geo-cultural backgrounds and values. Although our study did not utilize longitudinal mediation analysis—the typical gold standard of mediation designs—a recent comparison with cross-sectional designs—as applied in the present study—demonstrated that these latter designs offer a high level of power and well-managed Type I error rates, thereby offering a reasonable alternative when the exceedingly high number of data points required by longitudinal analysis is not practical [65].

A limitation of this study was the moderate sample size. In a sexually restricted society as Pakistan, it remains a challenge not only to recruit participants for a study on a taboo topic but, despite the guarantee of anonymity, to have them admit to pornography use and, in some instances, to pornography use that is problematic. Our confidence in the validity of the data stems from the use of best practices in survey development and implementation, the high internal consistency in assessed scores, and the alignment of substantial portions of our data with the existing literature/model on this topic. We further note we calculated the necessary sample size for this study prior to the data collection process. Specifically, the necessary sample size for α = 0.05 and β = 0.20 (the standards for scientific research), assuming 6 variables within a serial mediation analysis and a weak to moderate effect size, was 250 [66]. Our sample size of 280 exceeded this minimum by 30 participants. Second, although we followed best practices for online survey distribution and collection of data, research strategies that rely heavily on public and social media for recruitment are subject to biases in education, class, social media access, and other factors. Third, our study benefits from the fact that it was not a clinical sample of individuals seeking treatment. Such samples typically represent highly biased groups with specific and strong motivations (e.g., to overcome the stigma associated with treatment in Pakistan) that may differentiate them from more typical pornography users in Pakistan. At the same time, a clinical sample might have offered better insight into the etiological development of PPU. Fourth, although our findings support an ordered relationship from predisposing variables and craving to PPU via dysfunctional coping and lack of inhibitory control, longitudinal mediation analysis is necessary to verify these ordered effects and to help establish causal links rather than mere associations [67]. Indeed, such factors as anxiety and self-esteem undoubtedly represent perpetuating cycles where temporary relief from negative emotions is accompanied by longer-term anxiety as unwanted pornography cravings re-emerge. Such distinctions may be better explored via future intervention and/or qualitative analyses. Finally, we assessed only “stimulus-specific inhibitory control” whereas a more comprehensive view of the situation could be attained by simultaneous assessment of “general self-control,” as delineated in I-PACE [22]. While individuals may lack control in the presence of the addictive stimuli (e.g., pornography use), they may still have good general control over their behavior, a pattern that could be explored through comparison of problematic vs. non-problematic users. Specifically, problematic pornography users may lack both general and stimulus-specific control, whereas functional users may demonstrate either (or both) general or stimulus-specific control.

## 7. Conclusions

This study has delineated the pathway between predisposing factors and PPU through serial mediation of craving and coping/control, with mediating pathways accounting for nearly 30 to 40% of the variance in PPU, a highly respectable level for a study of this nature [68]. Furthermore, this study—using a behavioral addiction framework based on the I-PACE model—helps contextualize frequent/excessive pornography use as a disorder of cognitive/affective processes rather than as a deficiency of moral character, a common interpretation in social systems characterized by strong religious proscriptions regarding issues surrounding sexuality (e.g., masturbation, extramarital sex, etc.). Finally, this study has identified both points and strategies for interventions aimed at helping men and women who are distressed by their frequent pornography use, and in doing so, directly addresses aspects of psychological health in populations of younger men and women feeling out of control and/or socially marginalized by their unwanted cravings. An important challenge is that of not only identifying individuals in need but also lowering barriers to seeking help by de-stigmatizing use of mental health services in geo-cultural regions where shame and guilt are often associated with the use of psychological health facilities.

## Figures and Tables

**Table 1:** Descriptive characteristics of study participants.

Characteristics of Variable	*f*	*%*
Age		
18–28	191	68.2
29–39	88	31.4
40–50	1	0.4
Gender		
Female	123	43.9
Male	156	58.7
Sexual Orientation		
Bisexual	78	27.9
Homosexual	28	10.0
Heterosexual	174	62.1
Time Duration		
30 min or less	142	50.7
Up to 1 h	70	25.0
Between 1–2 h	48	17.1
3 or more hours	20	7.1
PV from How Long		
From last week	4	1.4
From last month	8	2.9
From last 5–6 months	2	0.7
From last year	15	5.4
From more than one year	251	89.6
Gratification Received		
It never makes me feel good	58	20.7
Sometimes	154	55.0
Most of the times	68	24.3

Note. PV = Pornography viewing, *ƒ* = frequency; *%* = percentage.

**Table 2:** Descriptive values and alpha coefficient of administered constructs (N = 280).

Variables	M	SD	Min	Max	α
Brief Pornography Screening	7.2	2.10	0	10	0.89
Craving (PCQ)	57.24	16.05	12	71	0.89
Dysfunctional coping (HBI-19)	20.49	5.14	7	35	0.91
Loneliness	3.40	1.96	0	6	0.81
Depression (DASS-21)	10.71	4.49	0	21	0.81
Stimulus-specific inhibitory control (HBI-19)	25.80	7.09	8	40	0.95
Self-esteem	28.06	5.37	10	40	0.92
Anxiety (DASS-21)	10.79	4.36	0	21	0.89

Note. PCQ = Pornography craving questionnaire, HBI-19 = Hypersexual Behavioral Inventory, DASS-21 = Depression, Anxiety, Stress Scale.

**Table 3:** Pearson correlation table of constructs administered (N = 280).

Variables	1	2	3	4	5	6	7	8
1.BPS	-							
2.CRV	0.420 **	-						
3.COP	0.396 **	0.282 **	-					
4.LS	0.060 *	0.233 **	0.107 *	-				
5.DEP	0.332 **	0.447 **	0.300 **	0.353 **	-			
6.AS	0.066 *	0.064 *	0.136 *	0.037 *	0.146 *	-		
7.SE	0.210 **	0.367 **	0.085 *	0.321 **	0.651 **	0.118 *	-	
8.CON	0.502 **	0.238 **	0.642 **	0.141 *	0.496 **	0.155 **	0.306 **	-

Note. BPS = Brief pornography screening, CRV = Craving, COP = HBI-19- dysfunctional coping, LS = loneliness, DEP = depression, AS = anxiety, SE = Self-esteem, CON = HBI-19- Stimulus-specific inhibitory control * *p* < 0.05, ** *p* < 0.01 (2-tailed).

**Table 4:** Sex/gender differences on major study variables (N = 280).

	Female (n = 123)		Male (n = 157)			
Variables	M	SD	M	SD	Cohen’s d	*p*
Loneliness	3.47	1.90	3.34	2.01	0.001	0.571
Self-Esteem	28.29	4.73	27.89	5.83	0.001	0.530
Depression	10.95	4.05	10.53	4.80	0.002	0.435
Anxiety	10.96	4.05	10.65	4.59	0.001	0.556
Brief pornography screening	7.18	2.37	7.24	1.82	0.000	0.821
Craving	57.22	16.99	57.26	15.31	0.000	0.983
Dysfunctional Coping	20.62	4.99	20.38	5.25	0.001	0.704
Stimulus-specific inhibitory control	25.96	7.11	25.68	7.08	0.000	0.745

Note. M = Mean, SD = standard deviation.

**Table 5:** Evaluation of indirect effects and bias-corrected 95% confidence interval.

Indirect Pathways	Standardized Indirect Estimate		
	B	SE	*p*
DEP → CRV→ PPU	0.144	0.04	0.001
LON → CRV → PPU	−0.011	0.019	0.567
ANX → CRV →PPU	−0.088	0.032	0.002
EST → CRV →PPU	0.087	0.032	0.000
DEP → CRV → CON → PPU	0.017	0.007	0.001
ANX → CRV → CON → PPU	−0.013	0.006	0.002
EST → CRB → CON → PPU	0.009	0.004	0.000
DEP → CRV → COP → PPU	0.015	0.007	0.001
ANX → CRV → COP → PPU	−0.012	0.006	0.002
EST → CRV → COP → PPU	0.008	0.004	0.001

Note. DEP = depression (DASS-21), CRV = Craving, COP = dysfunctional coping (HBI-19), LON = loneliness, ANX = anxiety (DASS-21), PPU = problematic pornography use, CON = Stimulus-specific inhibitory control (HBI-19), EST = self-esteem, B = estimates, SE = bootstrap standard error, *p* = significance value. Coefficients and *p*-values represent the total indirect effects of each of the pathways listed.

## Data Availability

Data output files from the analyses are available from the first author upon request.

## Appendix A. Questionnaires used in the Study

**Table:**

Questionnaires	Sample Items
DASS-21 (depression)	I couldn’t seem to experience any positive feeling at all I felt that I had nothing to look forward to
DASS-21 (anxiety)	I felt scared without any good reason I was worried about situations in which I might panic and make a fool of myself
Rosenberg self-esteem	At times I think I am no good at all I feel 1do not have much to be proud of.
DeJong Gierveld Loneliness	I experience a general sense of emptiness. I miss having people around me.
Pornography Craving Questionnaire	The thought of watching pornography makes me sexually aroused. I will watch pornography as soon as I get the chance.
Hypersexual behavioral Inventory-19 (Dys-coping)	Doing something sexual helps me feel less lonely. Doing something sexual helps me cope with stress.
Hypersexual behavioral Inventory-19 (SS-Inhibitory-Control)	Even though I promised myself I would not repeat a sexual behavior, I find myself returning to it over and over again. My attempts to change my sexual behavior fail.
Brief pornography screening	You find yourself using pornography more than you want to. You find it difficult to resist strong urges to use pornography

## Appendix B. Typical Invitation and Instructions Provided to Participants

Hi there!
I am Khifza Bibi, student of MS Clinical Psychology at Bahria University. I am doing research on watching adult themed content or pornographic content among adults.
If you are over age 18 years and watch adult themed/pornographic content you can participate in the study. If you are interested/willing to take a survey on this topic, please click on given link to participate. It will take approximately 15 to 20 min. You are requested to read each question carefully before answering. If you find anything perplexing or confusing, you may send your query on email. I assure you that your identity will not be revealed, and the survey process itself does not collect any information that could reveal your identity.
You have full right to withdraw your participation during any stage of the research.
Your support and participation will be highly appreciated.
For queries: khifzarehman999@gmail.com
**Thanking participants**
Thank you for your precious time and efforts. Kindly submit the form by clicking next.
